# *Aspergillus fumigatus* Infection in Humans With STAT3-Deficiency Is Associated With Defective Interferon-Gamma and Th17 Responses

**DOI:** 10.3389/fimmu.2020.00038

**Published:** 2020-01-28

**Authors:** François Danion, Vishukumar Aimanianda, Jagadeesh Bayry, Amélie Duréault, Sarah Sze Wah Wong, Marie-Elisabeth Bougnoux, Colas Tcherakian, Marie-Alexandra Alyanakian, Hélène Guegan, Anne Puel, Capucine Picard, Olivier Lortholary, Fanny Lanternier, Jean-Paul Latgé

**Affiliations:** ^1^Université de Paris, Centre d'Infectiologie Necker Pasteur, IHU Imagine, Hôpital Necker-Enfants Malades, Assistance Publique- Hôpitaux de Paris (AP-HP), Paris, France; ^2^Unité des Aspergillus, Institut Pasteur, Paris, France; ^3^Institut National de la Santé et de la Recherche Médicale, Centre de Recherche des Cordeliers, Equipe-Immunopathologie et Immunointervention Thérapeutique, Sorbonne Université, Université Paris Descartes, Sorbonne Paris Cité, Paris, France; ^4^Unité de Parasitologie-Mycologie service de Microbiologie, Hôpital Necker-Enfants Malades, Assistance Publique- Hôpitaux de Paris (AP-HP), Université de Paris, Paris, France; ^5^INRA USC 2019, Unite Biologie et Pathogenicite Fongiques, Institut Pasteur, INRA, Paris, France; ^6^Service de Pneumologie, Hôpital FOCH, Suresnes, France; ^7^Service d'Immunologie Biologique, Hôpital Necker-Enfants Malades, Assistance Publique- Hôpitaux de Paris (AP-HP), Université de Paris, Paris, France; ^8^Laboratoire de Parasitologie-Mycologie, Centre Hospitalier Universitaire de Rennes, Rennes, France; ^9^Univ Rennes, INSERM, IRSET (Institut de Recherche en santé, Environnement et travail) – UMR_S 1085, Rennes, France; ^10^St. Giles Laboratory of Human Genetics of Infectious Diseases, Rockefeller Branch, Rockefeller University, New York, NY, United States; ^11^Génétique Humaine des Maladies Infectieuses, Hôpital Necker-Enfants Malades, INSERM U1163, Paris and Université de Paris, Imagine Institut, Paris, France; ^12^Centre d'étude des Déficits Immunitaires (CEDI), Centre de Référence des Déficits Immunitaires Héréditaires (CEREDIH), Unité d'Immuno-Hématologie, Hôpital Necker-Enfants Malades, Assistance Publique- Hôpitaux de Paris (AP-HP), Paris, France; ^13^Université de Paris, Paris and Institut Imagine, INSERM UMR1163, Paris, France; ^14^Institut Pasteur, CNRS, Centre National de Référence Mycoses Invasives et Antifongiques, Unité de Mycologie Moléculaire, UMR 2000, Paris, France

**Keywords:** signal transducer and activator of transcription 3 (*STAT3*), loss-of-function mutation, aspergillosis, innate/adaptive immunity, autosomal dominant hyper-IgE syndrome (AD-HIES), *Aspergillus*, IgE, IgG

## Abstract

In humans, loss-of-function mutation in the *Signal Transducer and Activator of Transcription 3 (STAT3)* gene is frequently associated with susceptibility to bacterial as well as fungal infections including aspergillosis, although its pathogenesis remains largely unknown. In the present study, we investigated the immune responses obtained after stimulation with *Aspergillus fumigatus* in STAT3-deficient patients. *A. fumigatus* conidial killing efficiencies of both monocytes and neutrophils isolated from whole blood samples of STAT3-deficient patients were not different compared to those of healthy controls. After stimulation with *A. fumigatus* conidia, lower concentrations of adaptive cytokines (IFN-γ, IL-17 and IL-22) were secreted by peripheral blood mononuclear cells from STAT3-deficient patients compared to those from healthy controls. Moreover, the frequency of IFN-γ and IL-17 producing CD4+ T cells was lower in STAT3-deficient patients vs. healthy controls. Among the STAT3-deficient patients, those with aspergillosis showed further lower secretion of IFN-γ upon stimulation of their PBMCs with *A. fumigatus* conidia compared to the patients without aspergillosis. Together, our study indicated that STAT3-deficiency leads to a defective adaptive immune response against *A. fumigatus* infection, particularly with a lower IFN-γ and IL-17 responses in those with aspergillosis, suggesting potential therapeutic benefit of recombinant IFN-γ in STAT3-deficient patients with aspergillosis.

## Introduction

In humans, *Signal transducer and activator of transcription 3* (*STAT3*) encodes for a transcriptional regulator, and is a member of the STAT-protein family. STAT3-protein is activated notably by IL-6 family cytokines that signal through gp130 receptors (1), and activated protein has been considered to be an important signal transducer as it evokes distinct responses in different cells (1, 2). STAT3-protein plays a key role in controlling inflammation and immunity, particularly by regulating the expression of acute phase effector-elements (3, 4). Loss-of-function mutations in the *STAT3* gene (STAT3-deficiency) leads to autosomal dominant hyper-immunoglobulin E syndrome (AD-HIES), a primary human immunodeficiency (5, 6). Immunopathology associated with STAT3-deficiency is complex, as this protein is involved in several immunological processes.

STAT3-deficiency has been reported to increase the susceptibility to microbial infections of the skin and lungs, in addition to multisystem disease including cutaneous involvement and developmental defects (5). Susceptibility of the patients harboring *STAT3* mutation to infections by *Staphylococcus aureus or Candida albicans* has previously been investigated (7); although antimicrobial activity of the neutrophils from STAT3-deficient patients were comparable to that from healthy individuals, they displayed a lower production of the cytokines IFN-γ and IL-17, defective production of CXCL8 and antimicrobial peptides (BD2 and BD3) by epithelial cells (8, 9). STAT3-deficient patients showed an increased susceptibility to pulmonary aspergillosis, especially when they had preexisting lung cavities (10). Analysis of the French National Cohort of 74 patients with STAT3-deficiency indicated that 13 (18%) of them had developed at least one episode of pulmonary aspergillosis (11); these episodes were either chronic [aspergilloma and chronic cavitary pulmonary aspergillosis (CCPA)], allergic (allergic bronchopulmonary aspergillosis, ABPA) or mixed forms. However, the immunological defects associated with aspergillosis in STAT3-deficient patients remain unknown.

The objective of our study was to investigate the immune defects associated with STAT3-deficiency upon encountering conidia, the asexual spores which act as the infectious morphotype produced by the ubiquitous fungal pathogen *Aspergillus fumigatus*. We investigated conidial phagocytosis-killing efficiencies of neutrophils and monocytes, and innate and adaptive immune responses of peripheral blood mononuclear cells (PBMCs) isolated from STAT3-deficient patient-blood samples. Moreover, we compared anti-conidial responses of PBMCs isolated from STAT3-deficient patients with and without existing aspergillosis. Our major observation was that STAT3-deficiency is associated with lower adaptive immune responses against *A. fumigatus* infection.

## Materials and Methods

### Patients, Their Blood/Serum Samples

STAT3-deficient patients are followed in France by the Centre de Référence des Deficits Immunitaires Héréditaires (CEREDIH, Paris, France). We first collected sera from 32 patients with STAT3-deficiency to study IgE and IgG responses. We then included 12 STAT3-deficient patients, followed at Necker-Enfants Malades University Hospital, Paris France, for immunological study. Institutional review board approval was obtained (Comité de Protection des Personnes Ile de France 2, France, May 4th, 2015) and written consent was obtained from all the patients included in this study. Control samples were obtained from healthy donors [Etablissement Francais du Sang (EFS), Paris, France, habilitation HS-2015-25101].

Sera from patients with chronic pulmonary aspergillosis (CPA, *n* = 10; four patients had sarcoidosis, one lung cancer, one chronic obstructive pulmonary disease (COPD) and one sequelae following acute respiratory distress syndrome; underlying diseases were not known for the others) and allergic bronchopulmonary aspergillosis (ABPA, *n* = 11; four patients had cystic fibrosis and one asthma; for the others, underlying diseases were not known) and patients without STAT3-deficiency treated with substitutive intravenous immunoglobulins (*n* = 5) were recruited from Necker-Enfants Malades Hospital, Paris, and University Hospitals of Rennes and Lille, all in France.

### Isolation of Peripheral Blood Mononuclear Cells (PBMC), Monocytes and Neutrophils

PBMCs were separated on Lymphocytes Separation Medium (Eurobio) by density centrifugation of heparinized blood from STAT3-deficient patients or healthy controls, washed two times and re-suspended in RPMI 1640 + GlutaMAX (Gibco) supplemented with 10% of Normal Human Serum (NHS) and 1% of Pen-Strep (Gibco). PBMC count was determined using LUNA Automated Cell Counter with fluorescent dye to determine absolute number of live cells. Monocytes were purified from PBMC by positive-CD14 selection using CD14 MicroBeads with MS MACS columns (MACS, milltenyi biotec) following the protocol of the manufacturer. The purified monocytes were then resuspended in RPMI 1640 + GlutaMAX supplemented with 10% of NHS or autologous serum and 1% of Pen-Strep, and counted. Neutrophils were purified form the whole blood samples of STAT3-deficient patients and healthy controls using Neutrophil Isolation Kit (EasySep, Stemcell technologies) following the protocol provided by manufacturer; isolated neutrophils were re-suspended in RPMI 1640 + GlutaMAX with 0.5% NHS, and counted.

### *A. fumigatus* Conidia

*A. fumigatus* strain used in this study was CEA17ΔakuB^KU80^ that originates from the clinical isolate, CBS 144–89 (12). This strain was maintained on 2% malt-agar slants at ambient temperature. Conidia were harvested from 12 to 15-day old slants using 0.05% Tween–water, washed three times and resuspended in 0.05% Tween–water and then counted using LUNA Automated Cell Counter.

### FITC-Labeled Conidia

Conidia were incubated with fluorescein isothiocyanate (FITC; 0.1 mg/mL) in carbonate buffer (0.1 M, pH = 9) at 37°C in a shaken incubator for 1 h, washed three times with carbonate buffer and suspended in phosphate buffered saline (PBS).

### Para-formaldehyde (PFA) Fixed Swollen Conidia

Swollen conidia were obtained upon incubating 1 × 10^8^ dormant conidia strain in 50 mL RPMI at 37°C in a shaken incubator for 5 h. Swollen morphotype was verified by microscopy before collecting and washing with water. Conidia were then mildly sonicated to separate aggregates and then *para*-formaldehyde (PFA) fixed overnight at 4°C. Conidia were then washed three times with 0.1 M NH_4_Cl, one time with PBS, re-suspended in 1 mL of PBS and conserved at 4°C in aliquots for PBMC stimulation experiments. At least three different batches of fixed swollen conidial samples were used for PBMC stimulation experiments.

### Phagocytosis by Monocytes

After isolation, monocytes seeded in 96-well cell-culture plate (2 × 10^5^ per well) were added with 2 × 10^5^ FITC-labeled conidia suspended in RPMI 1640 culture medium containing 10% NHS and 1% Pen-Strep (total culture volume per well, 200 μL). After 1 h incubation, culture medium was removed; Calcofluor White (CFW; 5 μg/mL) in 200 μL of RPMI was added into each well to stain the non-phagocytosed conidia and incubated at room temperature for 15 min. After removing the media, monocytes were fixed with PFA (2.5%) overnight at 4°C. Phagocytosis was evaluated by counting intracellular (FITC only; green) and extracellular (CFW-labeled; blue) conidia under microscope (EVOS cell imaging system, Thermo Fisher Scientific) (13). At least 100 conidia were counted, in triplicate, and phagocytic percentage was expressed as a ratio between phagocytosed conidia out of all conidia counted.

### Conidial Killing Experiments

(i) *By monocytes –* performed using conidia of *A. fumigatus* parental strain, CEA17ΔakuB^KU80^. Monocytes (2 × 10^5^/ well) were incubated with 200 μL of culture medium containing 4 × 10^4^ conidia in 96 well culture-plates. After 3 h of co-incubation, wells were washed with PBS (3×) and fresh culture medium was added. After 8 h of co-incubation, supernatant was removed, wells were washed and cells were lysed with 400 μL of cold water. Media were collected and conidia were spread over Sabouraud-agar plate. Colony forming units (CFU) on the plates were counted after 36 h of growth at 37°C. Experiments were performed in triplicate. (ii) *By neutrophils*: Neutrophils [2.5 × 10^4^ (for MOI 0.5), 5 × 10^4^ (MOI 1), and 10 × 10^4^ (MOI 2)] were incubated with 5 × 10^4^ conidia for 15 h at 37°C in 200 μL of RPMI 1640 + GlutaMAX (Gibco) supplemented with 1% of Pen Strep and 0.5% NHS (Gibco). Conidia of CEA17ΔakuB^KU80^ strain were un-opsonized or opsonized with 20% of NHS in HEPES buffer for 30 min before adding to neutrophils. After co-incubation, supernatant was removed. Cells were lysed with cold water for 20 min and inhibition of germination (conidial killing) was evaluated with resazurin method (14, 15), a colorimetric assay that measures metabolically active fungus. Briefly, 30 μL of resazurin (0.1 mg/mL) was added with 100 μL of RPMI, incubated at 37°C for 48 h and the optical density (OD) was measured at 600 nm. If the fungus is metabolically active (alive), then resazurin changes from blue to pink in color due to the conversion of resazurin into resorufin (7-Hydroxy-3H-phenoxazin-3-one); the percentage of growth was evaluated measuring OD of the sample and comparing with that of positive control (live conidia not co-incubated with neutrophils) and from a negative control (without fungus or cells).

### PBMC Stimulation With *A. fumigatus*

To the PBMC (2 × 10^5^/well in 100 μL complete culture medium containing RPMI 1640 + GlutaMAX containing 10% NHS and 1% of Pen-Strep) in a 96-well culture plate added swollen-fixed conidia (2 × 10^5^ in 100 μL complete culture medium); culture medium alone was added to the control wells. Culture plates were then incubated at 37°C in a CO_2_ incubator for 1 or 5 days. Following, supernatant from the wells were collected and analyzed for cytokines (one-day culture supernatant for TNF-α, IL-1β, IL-6, IFN-γ, IL-8, and IL-10 and five-days culture supernatant for IFN-γ, IL-4, IL-5, IL-17A, IL-22, IL-6, IL-10) using ELISA duo-SET^®^ (R&D systems). After five-days culture, cells were collected, washed two times with RPMI, suspended in complete culture medium to have a cell count of 5 × 10^5^/mL and stimulated with phorbol myristate acetate (PMA) (50 ng/mL/0.5 million cells) and ionomycin (500 ng/mL/0.5 million cells), along with GolgiStop for 4 h (16). For the analysis of CD4 T-cell polarization (Th1, Th2, Th17, and Treg), surface staining was performed with fluorescence-conjugated MAbs to CD4, CD127, and CD25. Fixable viable dye was used to exclude dead cells. Cells were then fixed, permeabilized using an intracellular staining kit (eBioscience), and incubated at room temperature with fluorescence-conjugated MAbs to FoxP3, IFN-γ, IL-4, and IL-17A. Samples were processed further for flow cytometric analyses (LSR II, BD Biosciences). FITC-labeled anti-human IFN-γ, CD25-FITC, IL4-APC, CD127, BV421 antibodies were purchased from BD Biosciences; IL-17-PE and Fixable Viability Dye eFluor 506 from eBioscience; CD4-PerCP/cyanine5.5 from BioLegend; FoxP3-APC from Invitrogen. Data were analyzed by BD FACS DIVA (BD Biosciences).

### Immunoglobulin Quantification

Total IgE and specific anti-*Aspergillus fumigatus* IgE (m3) were measured using immunoCAP^®^ (Thermo Fisher Scientific) following manufacturer's instructions. Specific IgG were determined using three recombinant proteins from *A. fumigatus* [88 kDa, 18 kDa and catalase (17)]. These recombinant proteins were coated (5 μg/mL, 100 μL/well) in 96-well plates overnight at ambient temperature. After washing the wells with phosphate buffered saline (PBS)-Tween 0.05% and blocking with PBS-BSA (1%) for 1 h, sera were added (at 1:500 dilution) to the wells and incubated for 2 h, washes three times with PBS-Tween 0.05%, followed by the addition of horseradish-peroxidase conjugated anti-human IgG (A8667; Sigma-Aldrich) and incubating at ambient temperature for 1 h. After washing three times with PBS-Tween 0.05%, the reaction was developed using *O*-phenylene diamine (OPD), arrested with 4% H_2_SO_4_ and read at 492 nm.

### Statistical Analysis

Performed by one-way variance to compare three groups and Mann-Whitney test for two groups using GraphPad Prism-6.0, GraphPad software (La Jolla California USA).

## Results

### Description of the Patients

Twelve patients harboring loss-of-function *STAT3*-mutation (STAT3 deficiency) followed at CEREDIH, Necker-Enfants Malades Hospital, Paris, were included in this study. Their age was in the range of 20–55 years, and 67% of them were male. Characteristics of these patients and mutations are detailed in Table 1. Neutrophil and monocyte counts were normal in all patients. Immunophenotyping showed lower counts of T cells in one patient, memory B cells in seven patients and NK cells in seven patients (Table 1). Among 12 patients, six developed at least one episode of aspergillosis, including aspergilloma, chronic cavitary pulmonary aspergillosis (CCPA) and allergic bronchopulmonary aspergillosis (ABPA) (11). Three *Aspergillus* infections were ongoing at the time of this study. Six-patients displayed heterozygous *STAT3-*R382W mutation. No statistical difference in terms of age, sex, or immunophenotyping was evidenced between the six patients who developed aspergillosis (STAT3-asp) and those who did not (STAT3-w/o-asp).

**Table 1:** Characteristics of STAT3-deficient patients in this study.

**Age (years, range)**	***STAT3* mutation**	**Domain of mutation**	**Aspergillosis (onset)**	**Current Asp status**	**Other fungal infection**	**Antifungal treatment**	**Other treatment**	**CD4^**+**^/μl (460–1,232)**	**CD8^**+**^/μl (187–844)**	**CD19^**+**^/μl (92–420)**	**CD27^**+**^/CD19^**+**^ % (9–19)**	**NK/μl (89–362)**
30–39	R382W	DNA binding	CCPA (1992) ABPA (2012)	Prior Prior	No	Itraconazole	SC IgG Cloxacillin Aerosol colistine	1,508	624	286	10	104
20–29	R382W	DNA binding	ABPA (2010)	Ongoing	No	L-AmB 2 × /week	SC IgG Omalizumab TMP-SMZ, Azithromycin	1,250	600	325	10	175
30–39	R382W	DNA binding	Aspergilloma (1995)	Prior	Cutaneous fusariosis	Posaconazole	IV IgG Cloxacillin	525	360	315	3	45
20–29	R382W	DNA binding	ABPA (2007)	Prior	MCC	Posaconazole	IV IgG TMP-SMZ	760	380	304	3	152
20–29	R382W	DNA binding	No		MCC	Itraconazole	IV IgG TMP-SMZ	924	546	546	5	42
30–39	R382W	DNA binding	No		Cutaneous fusariosis	Itraconazole	TMP-SMZ, Azithromycin	142	191	142	2	42
20–29*	S560del	Linker domain	Aspergilloma (2011)	Ongoing	MCC	Itraconazole	SC IgG	588	384	72	11	84
30–39*	S560del	Linker domain	No		MCC	None	Cloxacillin	891	1269	243	4	189
50–59*	S560del	Linker domain	No		MCC	Fluconazole	None	700	364	140	11	98
20–29	V637M	SH2	ABPA (2005) CCPA (2013)	Prior Ongoing	No	Posaconazole	IV IgG Aerosol AmB TMP-SMZ, Azithromycin	774	396	306	6	72
30–39	I568F	Linker domain	No		MCC	None	IV IgG	918	450	360	9	36
20–29	S668Y	SH2	No		No	Itraconazole	IV IgG Cloxacillin	855	240	225	8	45

* *Relatives of the same family. CCPA, chronic cavitary pulmonary aspergillosis; ABPA, allergic bronchopulmonary aspergillosis; Asp, aspergillosis; MCC, Mucocutaneous Candidiasis; L-AmB, liposomal amphotericin B; IgG, immunoglobulin G; SC, subcutaneous; IV, intravenous; TMP-SMZ, trimethoprim-sulfamethoxazole*.

### STAT3-Deficient Patients With Aspergillosis Showed High Amount of Specific Anti-*aspergillus* IgE and IgG

We had access to the sera from 32 STAT3-deficient patients from the French national cohort (including 12 patients in this study). Eleven of them had developed aspergillosis (STAT3-asp: six with chronic pulmonary aspergillosis (CPA), three allergic bronchopulmonary aspergillosis (ABPA), and two had both ABPA-CPA; eight were with ongoing aspergillosis and three in complete remission at the time of our study). We compared them with healthy controls, patients with ABPA or CPA (without STAT3-deficiency) and patients supplemented with intravenous immunoglobulin.

**Figure 1:**
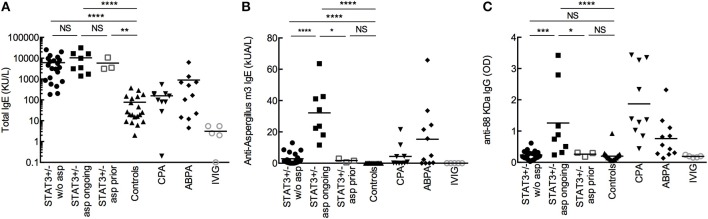
Total IgE **(A)**, specific IgE **(B)**, and IgG **(C)** against *A. fumigatus*. Comparison of sera from STAT3 deficient-patients (STAT3^+/−^, representing heterozygous mutation) without aspergillosis (w/o asp; *n* = 21), with ongoing aspergillosis (asp ongoing; *n* = 8) or with prior aspergillosis (asp prior; *n* = 3), with healthy controls (*n* = 20), CPA (*n* = 10), and ABPA (*n* = 11) patients, and patients receiving substitutive intravenous immunoglobulin (IVIG; *n* = 5). OD: optic density; *p < 0.05, ** *p* < 0.01, *** *p* < 0.001, and **** *p* < 0.0001 (the mean values are presented in the figures).

Among the STAT3-deficient patients, all of them (*n* = 32; 100%) showed increased total IgE titers (>114 kU/L) and in 29 patients (91%) specific anti-*Aspergillus* IgE was detected (≥0.1 kUA/L) (Figures 1A,B, Table 2). STAT3-deficient patients with ongoing aspergillosis had higher serum levels of specific anti-*Aspergillus* IgE titers than STAT3-deficient patients without aspergillosis (*p* < 0.01), whereas no difference in total IgE was observed. Specific IgG against three recombinant proteins of *A. fumigatus* [88, 18 kDa, and catalase] were significantly higher in STAT3-deficient patients with ongoing aspergillosis compared to those without aspergillosis (representative data shown for *A. fumigatus* 88 KDa proteins in Figure 1C and Table 2; *p* < 0.01). We then used a combination of three criteria including total IgE > 1,000 kU/L (the cut-off used for ABPA diagnosis), specific anti-*Aspergillus* IgE ≥ 0.1 and specific anti-*Aspergillus* IgG (88KDa; titer above the value of 97.5 percentile of the healthy controls was used as cut-off). Five (62.5%) STAT3-deficient patients with ongoing aspergillosis had these three positive tests compared to none in the STAT3-deficient patients without or with prior aspergillosis (*p* < 0.01).

**Table 2:** Total IgE and specific anti-*Aspergillus* IgE and IgG based on aspergillosis phenotype.

**Controls/patients**	**N**	**Total IgE kU/L Median (range)**	**Total IgE > 1,000 *N* (%)**	**Asp-IgE kUA/L Median (range)**	**Asp-IgE > 0.1 *N* (%)**	**Asp-IgG 88kDa Optic density Median (range)**	Asp-IgG > cut-off*N (%)	Triple positive criteria **N (%)
STAT3-deficient patients	32	3,527 (182–31,302)	26 (81%)	2.1 (<0.1–63.6)	29 (91%)	0.29 (0.03–3.42)	5 (16%)	5 (16%)
Without aspergillosis	21	3,579 (182–25,472)	15 (71%)	1.3 (<0.1–13)	18 (86%)	0.22 (0.03–0.62)	0	0
With ongoing aspergillosis	8	6,683 (1,397–31,302)	3 (100%)	29.0 (11.6–63.6)	8 (100%)	0.81 (0.24–3.42)	5 (63%)	5 (63%)
CPA	4	10,003 (1,397–31,302)	4 (100%)	33.2 (11.6–63.6)	4 (100%)	0.95 (0.24–3.42)	3 (75%)	3 (75%)
ABPA	2	5,808 (1,721–9,894)	2 (100%)	37.8 (34.2–41.1)	2 (100%)	0.60 (0.31–0.88)	1 (50%)	1 (50%)
CPA and ABPA	2	9,665 (3,108–16,222)	2 (100%)	20.2 (18.1–22.3)	2 (100%)	1.65 (0.51–2.80)	1 (50%)	1 (50%)
With prior aspergillosis	3	3,474 (3,106–10,801)	3 (100%)	1.6 (0.9–2.9)	3 (100%)	0.30 (0.18–0.31)	0	0
Healthy controls	20	22.5 (2–374)	0	<0.1	0	0.15 (0.05–0.92)	1 (5%)	0
CPA	10	125 (0.2–545)	0	0.5 (<0.1–21.5)	8 (80%)	1.38 (0.44–3.43)	9 (90%)	0
ABPA	11	163 (4.5–6311)	2 (18%)	4.9 (<0.1–65.8)	8 (73%)	0.55 (0.13–2.32)	5 (45%)	1 (9%)
Substitutive IVIG	5	2.5 (0–5.5)	0	<0.1	0	0.19 (0.15–0.24)	0	0

*N = number of patients. Asp-IgE, specific anti-Aspergillus IgE; Asp-IgG, specific anti-Aspergillus IgG; CPA, chronic pulmonary aspergillosis; ABPA, allergic broncho-pulmonary aspergillosis*;

* *Specific anti-Aspergillus IgG cut-off defined by the value of 97.5 percentile of the healthy controls*.

** *Triple positive criteria defined by total IgE > 1,000 kU/L, specific anti-Aspergillus IgE > 0.1 kUA/L and specific anti-Aspergillus IgG > cut-off*.

### Innate Immune Cells From STAT3-Deficient Patients Display a Normal Clearance of *A. fumigatus* Conidia

**Figure 2:**
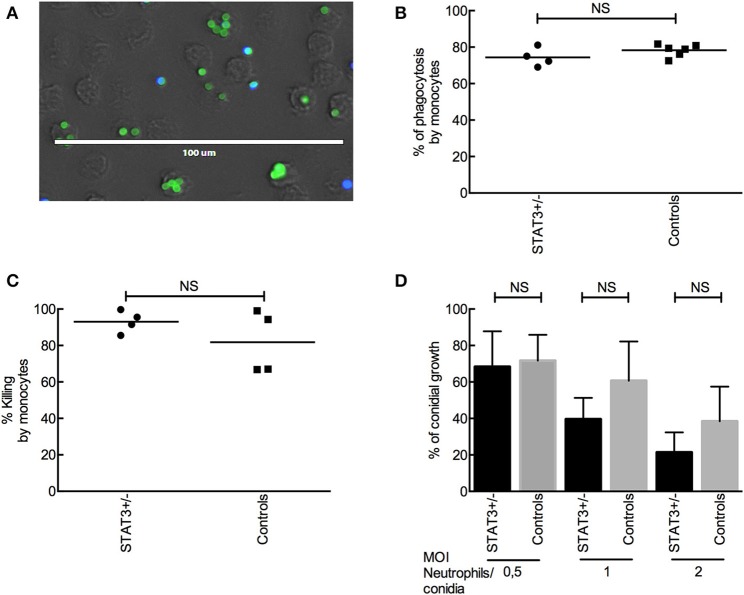
*A. fumigatus* conidial phagocytosis and killing by monocytes and neutrophils. **(A)** Phagocytosis of FITC-labeled conidia (parental strain) by CD14^+^ monocytes from STAT3-deficient patient (STAT3^+/−^) and controls. Extracellular conidia are labeled with CFW (in blue) whereas intracellular conidia are pre-FITC-labeled (in green). **(B)** Phagocytosis of Δ*ku80* conidia by CD14^+^ monocytes from four STAT3-deficient patients and six healthy controls. **(C)** Killing of Δ*ku80* conidia by CD14^+^ monocytes from four each of STAT3-deficient patients and healthy controls, and evaluated by colony forming unit (CFU) counting. **(D)** Killing of Δ*ku80* conidia by neutrophils at different conidia: neutrophils ratios from six each of STAT3-deficient patients and healthy controls.

Phagocytic and killing capacities of innate immune cells from STAT3-deficient patients were investigated. There were no significant differences in the phagocytosis-killing of *A. fumigatus* conidia by CD14^+^ monocytes isolated from whole blood samples of STAT3-deficient patients and healthy controls (Figures 2A–C). Also, conidial killing by the neutrophils isolated from whole blood samples of STAT3-deficient patients was similar to that of healthy controls (Figure 2D). Further categorization of STAT3-deficient patients into those with or without aspergillosis did not show any significant difference in the conidial phagocytosis as well as killing between these two groups, or compared with healthy controls.

### STAT3-Deficient Patients Exhibit Low Th17 and IFN-γ Responses to *A. fumigatus*

**Figure 3:**
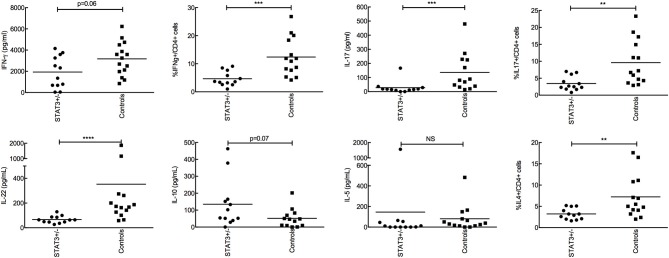
Analysis of adaptive immune response after stimulating PBMCs with conidia for 5-days. 2 × 10^5^ conidia were co-incubated with 2 × 10^5^ PBMC from STAT3-deficient patients (STAT3^+/−^) and healthy controls. Secretion of IFN-γ, IL-17, IL-22, IL-10, IL-5 were analyzed by ELISA and expressed in pg/mL, while, percent CD4+ T cells expressing IFN-γ, IL-17, IL-4 was analyzed by FACS; ** *p* < 0.01, *** *p* < 0.001, and **** *p* < 0.0001.

As no difference was evidenced in the phagocytic function, the secretion of cytokines by peripheral blood mononuclear cells (PBMC) following *A. fumigatus* conidial (swollen-PFA fixed) stimulation was investigated. The production of TNF-α, IL-1β, IL-6, IL-8, and IL-10 by PBMCs in response to one-day interaction with conidia (innate cytokine) was similar in STAT3-deficient patients compared with healthy controls (data not shown). Whereas, after 5 days of PMBCs-conidial interaction, the frequency of IL-17-producing CD4+ T cells (Th17) and the production of TH17 cytokines IL-17 and IL-22 were significantly lower in STAT3-deficient patients compared to healthy controls (Figure 3). Also, the frequency of IFN-γ-producing CD4+ T cells (Th1) and the amount of IFN-γ secreted in STAT3-deficient patients upon conidial stimulation of their PBMCS were decreased compared to healthy controls. Among Th2 cytokines, secreted IL-4 was not detected by ELISA and was lower in STAT3-deficient patients upon FACS analysis (intracellular), whereas IL-5, measured by ELISA, and Treg frequency (CD4^+^CD25^+^Foxp3^+^) measured by FACS were not different between STAT3-deficient patients and healthy controls (data not shown).

### Low Pro-inflammatory and IFN-γ Responses Are the Characteristic Features of STAT3-Deficient Patients With Aspergillosis

**Figure 4:**
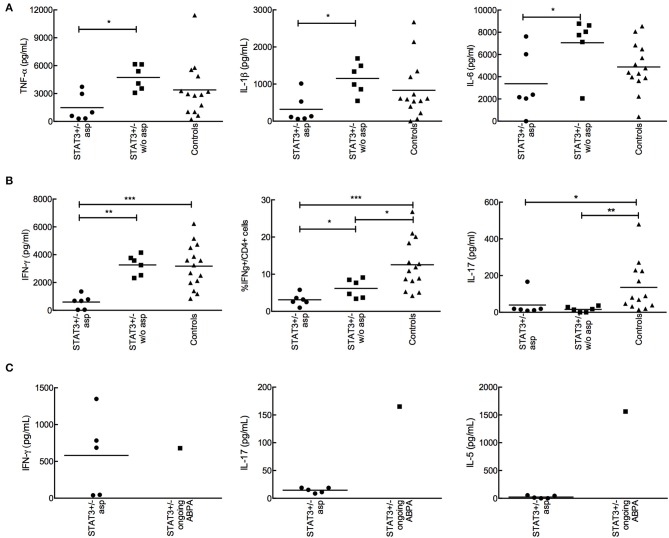
Analysis of the cytokine production by PBMCs isolated from STAT3-deficient patients with or without aspergillosis upon interaction with *A. fumigatus*. 2 × 10^5^ conidia were co-incubated with 2 × 10^5^ PBMC during 1 (innate response) or 5 days (adaptive response). **(A)** TNF-α, IL-1β, and IL-6 secreted by PBMCs isolated from STAT3-deficient patients (STAT3^+/−^) with aspergillosis (STAT3 asp) (*n* = 6), without aspergillosis (STAT3 w/o asp) (*n* = 6) and healthy controls upon one-day interaction with *A. fumigatus* conidia; cytokines were analyzed by ELISA. **(B)** IFN-γ and IL-17 secreted upon stimulation of PBMCs isolated from STAT3-asp, STAT3-w/o-asp and healthy controls with *A. fumigatus* conidia for 5-days; ELISA (secreted) and FACS (intracellular) were performed. **(C)** Analysis of IFN-γ, IL-17 and IL-5 secretion in one STAT3-deficient patient with ongoing ABPA compared to STAT3-deficient patient with aspergillosis (*n* = 5); *p < 0.05, ** *p* < 0.01, and ***p < 0.001.

No difference was observed in the innate cytokine levels between STAT3-deficient patients and healthy controls (Figure 3). However, when STAT3-deficient patients were categorized with and without aspergillosis (STAT3-asp and STAT3–w/o-asp, respectively), we observed that the secretion of pro-inflammatory cytokines by PBMCs upon interaction with *A. fumigatus* conidia, including TNF-α, IL-1β, and IL-6 (day-1) but also IFN-γ (day-5) were significantly lower in STAT3-asp compared to STAT3–w/o-asp patients (Figures 4A,B). In the subgroup of STAT3-deficient patients with aspergillosis, no difference in the IFN-γ, IL-6, TNF-α, and IL-1β secretion were observed among patients with ongoing vs. prior aspergillosis (data not shown). Of note, among STAT3-asp patients, one with ongoing ABPA showed higher secretion of IL-5 and IL-17 and low secretion of IFN-γ (Figure 4C).

## Discussion

Our study shows no intrinsic innate immune defects, such as phagocytosis and killing, in the patients harboring loss-of-function mutations in the *STAT3* gene (STAT3-deficiency), toward *A. fumigatus*. On the contrary, these STAT3-deficient patients showed a defective adaptive immune response, with lower production of cytokines including IFN-γ, IL-17, and IL-22. Moreover, those STAT3-deficient patients who developed aspergillosis showed further lower level of IFN-γ than the STAT3-deficient patients without aspergillosis. One major protective host mechanism against *A. fumigatus* infection is via Th1 and IFN-γ and a recent study showed that majority of lung-derived T cell phenotype was Th17 upon *A. fumigatus* infection (18, 19). These observations suggest that lower production of Th1 and Th17 cytokines in the STAT3-deficient patients could be the reason for their susceptibility to *A. fumigatus* infection.

STAT3-deficient patients with ongoing aspergillosis had higher specific anti-*Aspergillus* IgE and IgG titers compared to those without aspergillosis. The combination of three positive criteria (total IgE > 1,000 kU/L, specific anti-*Aspergillus* IgE ≥ 0.1 and specific anti-*Aspergillus* IgG above the cut-off) was associated with aspergillosis in the STAT3-deficient population. Further studies are warranted to confirm these results and to identify the adequate cut-off to be used in the diagnosis. We also noticed that substitutive IVIG, often used in STAT3-deficient population, did not falsely increase anti-*Aspergillus* IgG titers in a control population (Figure 1C, Table 2).

Total and specific anti-*Aspergillus* IgE are used in the diagnosis of allergic forms of aspergillosis (ABPA), notably in those patients with cystic fibrosis and asthma (20). This was also evidenced in chronic granulomatous disease (CDG) patients (21): elevated total (>1,000 kU/L) and specific anti-*Aspergillus* IgE were noticed in four out of eleven CGD patients. Our group has recently described a CGD patient with *Aspergillus felis* invasive infection who had elevated total and specific anti-*Aspergillus* IgE (22). In another study, a patient with CARD9 deficiency (due to homozygous mutation) and extra-pulmonary aspergillosis had an elevated total IgE level (23). Thus, elevated total (>1,000 kU/L) and specific anti-*Aspergillus* IgE can be observed in the setting of STAT3 deficiency, not only in patients with ABPA-like presentation as evidenced by Duréault et al. (11), but also in other forms of STAT3 deficiency-related pulmonary aspergillosis as evidenced here. In addition, such immunological features can also be observed in other primary immune deficiencies complicated by aspergillosis, including in patients without ABPA criteria. In STAT3-deficient patients an elevated total IgE level is characteristic of the disease, and possibly be explained by IL-21 signaling defect (24). The defective IL-10 (anti-inflammatory) response may lead to an exaggerated immune response resulting in allergic form of aspergillosis (25, 26).

The normal function of neutrophils of STAT3-deficient individuals against *Aspergillus* has already been shown earlier (10, 27); we confirmed these results in our study, and in addition we showed a normal function of monocytes from STAT3-deficient patients for phagocytosis and killing of *A. fumigatus* conidia. Also, innate cytokine production by PBMCs from STAT3-deficient patients was similar to that of healthy controls. Together, this absence of innate immune defect in the STAT3-deficient patients might explain why invasive aspergillosis is very rare in this primary immunodeficiency condition unlike in Chronic Granulomatous Disease (CGD) patients displaying a defective killing of *Aspergillus* by neutrophils, and these patients develop invasive aspergillosis (28, 29). Nevertheless, airway epithelial cells and alveolar macrophages from STAT3-deficient patients have never been studied for *Aspergillus* infection, but we are limited in obtaining these samples.

A defect in IFN-γ response has already been shown in STAT3-deficient patients upon stimulation with heat-killed *Staphylococcus aureus* and *Candida albicans*, two other major pathogens in STAT3-deficient phenotype (9, 30). We also observed a defect in the adaptive IFN-γ secretion from PBMC of STAT3-deficient patients following stimulation with *A. fumigatus*. This lower secretion of IFN-γ by PBMCs was contributed mainly from the STAT3-deficient patients with aspergillosis, and no difference in the IFN-γ secretion was evidenced between the patients with ongoing and prior aspergillosis. The lower secretion of IFN-γ and TNF-α by T cells was also reported upon phytohaemagglutinin (PHA) and anti-CD2/CD3/CD28 microbeads stimulation in AD-HIES patients (31, 32). Some other studies did not demonstrate any defect in IFN-γ production by STAT3-deficient patients (8, 24, 32). This may be explained by different stimuli used (staphylococcal enterotoxin and antigens of *Candida albicans* but not heat-killed pathogens) and by other immune cell studied (neutrophils).

IFN-γ secretion defect after *Aspergillus* stimulation in our cohort is of interest as IFN-γ is the cornerstone in defense against aspergillosis (33). It was shown that in patients with hematopoietic stem cell transplantation and hematological malignancy, an increased IFN-γ response to recombinant proteins of *A. fumigatus* cell wall was associated with improved outcome of invasive aspergillosis (34). Whereas, renal allograft recipients with invasive fungal infection failed to show any increase in the level of IFN-γ (35). Interestingly, mortality due to invasive aspergillosis in experimental murine infection model was related to an impaired IFN-γ response in mice (36), and IFN-γ therapy had a protective role (37). IFN-γ increases killing capacities of human neutrophils and monocytes against *A. fumigatus* hyphae and *A. terreus* and the release of pro-inflammatory cytokines (38, 39). Recombinant IFN-γ therapy was therefore used in several clinical trials to treat or at least to prevent fungal infections (33, 40–42). In a randomized trial with 128-CGD patients, IFN-γ therapy was an effective and well-tolerated treatment that reduced the frequency of serious infections and increased the ability of neutrophils to damage *Aspergillus* hyphae (40, 43). IFN-γ therapy was also able to partially restore immune function in a small open-label series of eight patients with invasive candidiasis and/or aspergillosis (36). HLA-DR expression and secretion of pro-inflammatory cytokines by leukocytes were increased following IFN-γ therapy. Moreover, IFN-γ treatment was efficient to inhibit IgE production in STAT3-deficient patients (44). In the French STAT3-deficient cohort, we identified three patients treated with combination of IFN-γ and antifungals for four episodes of aspergillosis, and two of them showed favorable outcome. Association of IFN-γ with antifungal treatment and surgery limits the specific evaluation of IFN-γ therapy but confirms the urgent need of further studies on IFN-γ therapy in the STAT3-deficient patients with aspergillosis.

We also showed a defect in secretion of IL-17 by PBMC and IL-17-producing CD4^+^ T cells after *A. fumigatus* stimulation. Defect in IL-17 has already been reported in STAT3-deficient patients after anti-CD3/CD28 monoclonal antibodies, *Candida* or *Staphylococcus* antigen stimulations, leading to a defective production of anti-staphylococcal factors (neutrophil-recruiting chemokines and antimicrobial peptides) by epithelial cells (8, 45). IL-17 defect explain a large part of *Candida* and *Staphylococcus* skin infections in this deficiency and may participate to *Aspergillus* susceptibility.

Of the 12 STAT3-deficient patients included in this study, six of them had mutation in the DNA-binding domain (R382W), four in the linker domain (three S560del and one I568F) and two others showed random mutations in the SH2 domain (V637M and S668Y) in the *STAT3* gene. The comparison of IFN-γ produced according to these mutations indicated that the mutation in DNA-binding domain resulted in a significant decrease in the IFN-γ produced compared to healthy controls (*p* < 0.01) and to patients with a *STAT3* mutation in the linker domain (*p* = 0.04). The difference of IFN-γ produced was not significant between healthy controls and the patients with a mutation in the linker domain or SH2 domain, suggesting that the type of *STAT3*-mutation, mainly in the DNA-binding domain, may impact on IFN-γ production. No difference was evidenced for other cytokines tested when grouped according to the mutation types.

To conclude, though loss-of-function mutations in *STAT3* gene is a rare primary immunodeficiency, and our study is limited by the number of STAT3-deficient patients, our data indicates that STAT3-deficient patients, particularly those with aspergillosis, exhibit adaptive immune defect by producing lower IFN-γ and Th17 responses toward *A. fumigatus* infection but not any defects in their innate immune functions. The presence of lung cavities in association with defective adaptive immune responses, defective production of antimicrobial peptides and chemokine might partially explain the development of pulmonary aspergillosis during STAT3-deficiency. This warrants innovative immunotherapeutic approaches, based on cytokine, to treat STAT3-deficient patients with severe chronic forms of pulmonary aspergillosis.

## Data Availability Statement

The datasets generated for this study are available on request to the corresponding author.

## Ethics Statement

The studies involving human participants were reviewed and approved by Comité de Protection des Personnes Ile de France 2, France, May 4th, 2015. The patients/participants provided their written informed consent to participate in this study. Written informed consent was obtained from the individual(s) for the publication of any potentially identifiable images or data included in this article.

## Author Contributions

FD, VA, JB, OL, FL, and J-PL contributed to the conception of the work. All authors contributed to the acquisition, analysis or interpretation of the data, manuscript revision, read, and approved the submitted version. FD wrote the first draft. VA, JB, OL, FL, and J-PL wrote sections of the manuscript. VA and SW edited the manuscript.

### Conflict of Interest

The authors declare that the research was conducted in the absence of any commercial or financial relationships that could be construed as a potential conflict of interest.
